# Sense of Belonging in Science: A Focus on the Construct

**DOI:** 10.1111/jedm.70038

**Published:** 2026-04-01

**Authors:** Linda Morell, Mingfeng Xue, Katherine Nielsen, Sabine Jeske, Rachel Harris, Michelle Phillips, Mark Wilson

**Affiliations:** University of California, Berkeley; University of North Carolina, Greensboro; University of California, San Francisco; University of California, San Francisco; University of California, San Francisco; Phillips & Associates; University of California, Berkeley

## Abstract

The importance of the construct to the fields of measurement and cognitive science cannot be overstated. Without an understanding of the construct, including clarity and scope of the substantive and structural aspects, one cannot ensure valid use and interpretation of a constructed measure. In this sense, measurement and cognitive science are fundamentally connected through the construct. One important construct that lacks clarification in definition and structure is a sense of belonging in science. This is especially true for adolescent age students. Unfortunately, this attribute is often vaguely conceptualized and largely missing from the literature for school-age students, and therefore, in need of a rigorous theoretical foundation supported by empirical evidence to ensure meaningful use of any assessment or interpretation of results. Using the BEAR Assessment System’s approach to constructing measures, which includes using Rasch family modeling, data were collected from high school students through eight exploratory interviews, 12 think-aloud interviews, focus groups comprised of 9–12 participants per group (45 students in total), and 537 surveys; and from five experts to develop and validate a 12-item self-report survey of high school students’ sense of belonging in science. Sources of evidence for reliability, validity (content, internal structures, response processes, and relation to other variables), and fairness were collected and analyzed to produce a construct-centered survey. Overall, findings produced strong evidence for the valid, reliable, and fair interpretation and use of the Sense of Belonging in Science Survey (BiSS). This research highlights the importance of the construct as the driver of the design and quality assurance of a measure.

## Introduction

The role of the construct in both measurement and cognitive science is critical. Accurately defining and understanding its substantive and structural scopes are essential for ensuring the valid use and interpretation of any constructed measure. Thus, the construct can serve as a link from the field of measurement to cognitive science. The construct acts as a conceptual anchor, guiding the design of measurement tools while being informed by cognitive science. This study focuses on the construct of sense of belonging in science and presents how using a construct-based approach to measurement ensures the development of an instrument that is well-designed, useful, and valid. In the study, we also emphasize the importance of integrating information from multiple sources to define and clarify the construct in the measurement development process, including but not limited to literature, targeted respondents, stakeholders, and social contexts.

### Definition

The current conception of educational or psychological “validity” began to be discussed in the 1950s when researchers theorized aspects of test validity and argued that “since predictive, concurrent, and content validities are all essentially ad hoc, construct validity is the whole of validity from a scientific point of view” (Loevinger, 1957, p. 636). However, a separated view of validity (e.g., content validity, construct validity, and criterion validity) persisted until Samuel Messick (1989) unified and integrated these aspects of validity into a more comprehensive theory of “construct validity.” In Messick’s (1989, 1994, 1995) seminal theoretical work on validity, he argued that the traditional view of validity was incomplete and fragmented. Therefore, he (1989) presented a more unified view of the concept of validity, which places greater emphasis on how an assessment is used, and reframes validity into six strands of validity evidence dependent on each other, supportive of each other, and not separate forms of validity evidence. Messick (1989) termed these six strands of construct validity: content, substantive, structure, generalizability, external factors, and consequential validity evidence. Messick’s ideas about validity have had a lasting impact on educational and psychological measurement as can be seen in a widely used guide for assessment development (e.g., the Standards for Educational and Psychological Testing, 2014). Given Messick’s unifying work on the concept of construct validity, close attention needs to be given to the definition of a construct. Wilson (2023, p. 9) suggests that “a construct could be a part of a theoretical model of a person’s cognition, such as their understanding of a certain set of concepts, or their attitude toward something, or it could be some other psychological” attribute such as extraversion, sense of belonging, or some other idea that “ideally, [has] a theoretical structure.” The theoretical structure that Wilson identifies can be considered the construct and can be investigated through Messick’s conception of construct validity.

For some in measurement, the psychometric model can take prominence over other aspects of validation. But as Messick (1989) and others (Morell et al., 2024; Wilson, 2023) suggest, the validation quest requires the integration of the conceptual model, items, the meaning/interpretation of scores (or valuation), along with a psychometric analysis with the basis for all being the conceptual model—also known as the theory or construct.

### Approaches

Researchers in education and psychology have proposed many frameworks to embody the construct in measurement. Four commonly used frameworks are the National Research Council’s Assessment Triangle (Glaser et al., 2001), an argument-based framework (Kane, 2013), Evidence Centered Design (Mislevy et al., 2003), and the Berkeley Evaluation & Assessment Research (BEAR) Assessment System (Wilson, 2023).

#### Berkeley evaluation and assessment research (BEAR) assessment system.

**Figure 1.**
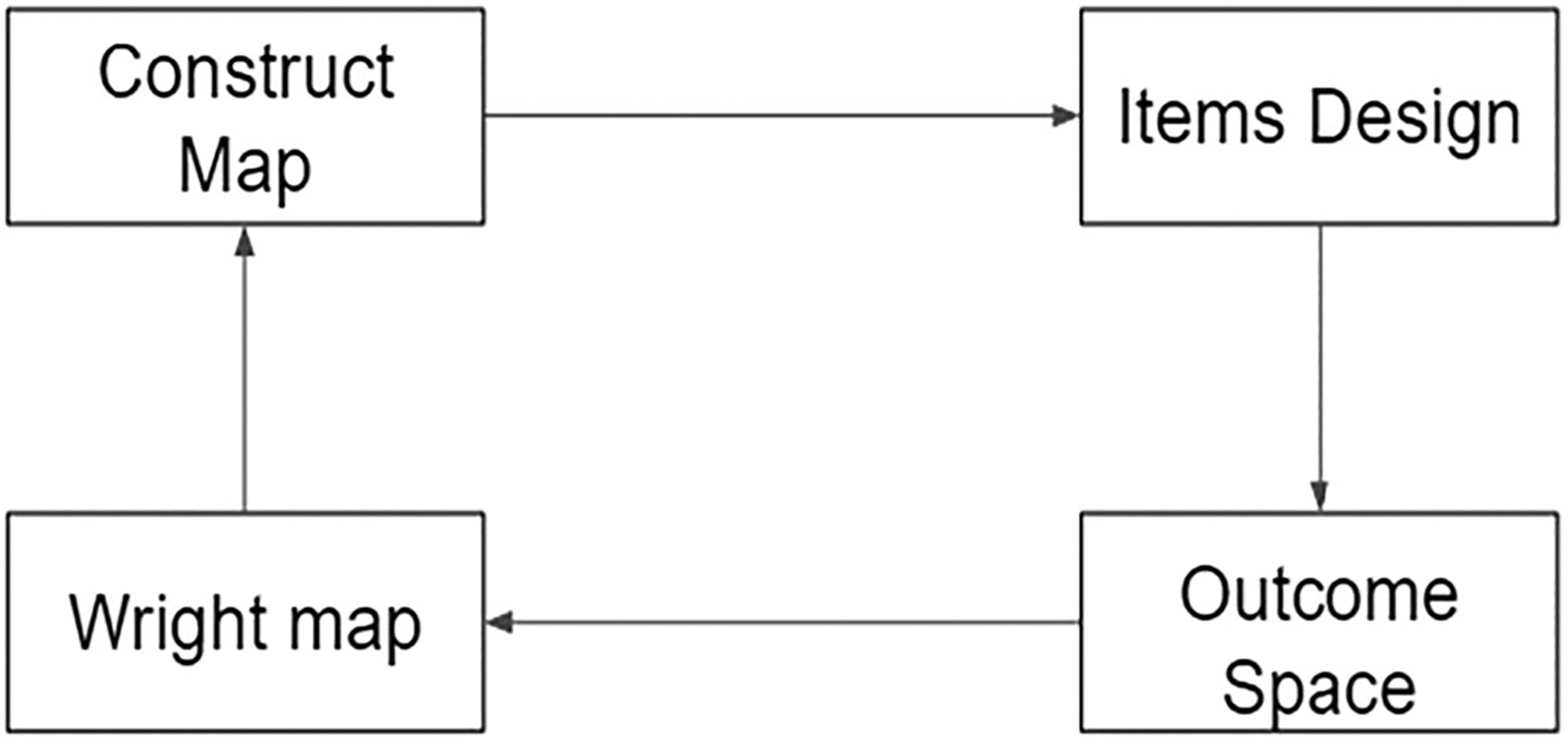
The four building blocks of BEAR Assessment System.

The BEAR Assessment System (BAS) places the construct at the center of the assessment development process. Therefore, it was chosen as the assessment framework for this study. The BAS consists of four building blocks (see Figure 1) that embody the construct in assessment (Wilson, 2023). The framework starts with a construct map, which outlines the definition of the construct and defines several qualitatively different and ordinal levels (aka waypoints). In the next building block, items are designed to assess each waypoint, while in the outcome space building block, students’ responses are classified into the different waypoints, and scoring guides are often generated at this step. The Rasch family of models are used to model the relationship between observed responses and latent constructs. Wright maps, a visual aid generated from Rasch modeling, provide internal structure validity evidence for the construct map by showing students’ proficiency and item Thurstonian thresholds side-by-side. Note that Thurstonian thresholds are the locations on the continuum of construct where students have an equal chance of achieving at a certain waypoint or higher and represent the empirical difficulty of waypoints.

While often used to develop achievement-based assessments, the BEAR Assessment System can also be used to develop and validate measures of affective attributes. Using *a sense of belonging in science* as an example, we illustrate how the construct-oriented BAS framework can be used in this context.

### Sense of Belonging in Science—Background

Disparities in representation and opportunity persist in academic, clinical, and industry settings. Lack of racial and ethnic minority representation in the science-related workforce is, in part, a result of attrition of talented students from historically marginalized communities (often referenced in the literature as underrepresented minorities, URM) at each stage of the educational and career path. For example, students from backgrounds historically marginalized in science with an initial interest in science leave science majors and college at a higher rate than students from the non-minority group (Seymour & Hewitt, 1997; NRC, 2003; George et al., 2001). Contributing to this attrition is a phenomenon called “belonging uncertainty,” or lack of a sense of belonging to a community of practice or educational institution (Walton & Cohen, 2007). Belonging uncertainty develops in these students as many social inequities and environmental factors compound, including but not limited to attending workplaces or schools where few share their racial or ethnic identity (and in particular lack role models from similar backgrounds), encountering negative expectations from peers and faculty that contribute to stereotype threat, and feeling cut off from “insider” information that their white peers seem to enjoy (Walton & Cohen, 2007; Fisher et al., 2019).

The opposite of belonging uncertainty is perceived belonging, which correlates with gains in academic achievement, motivation, and sense of well-being (Anderman & Freeman, 2004; Freeman et al., 2007; Pittman & Richmond, 2007; Wilson et al., 2013). Moreover, there appears to be a link between historically minoritized students’ persistence in fields where they are underrepresented and a feeling of connectedness or sense of belonging to that field, underscoring that sense of belonging in science may be key to addressing the attrition of minority students in science, technology, engineering, and mathematics (Hausmann et al., 2007; Tinto, 1993; Astin, 1993). Prior research (Mahar et al., 2012) indicates that belonging encompasses a variety of factors such as acceptance, respect, representation, inclusion, and support.

Sense of belonging in science is deeply interconnected with science identity development (Carlone & Johnson, 2007; Trujillo & Tanner, 2014; Clark et al., 2016). Research on identity development has been strongly influenced by the work of Lave and Wenger, who describe identities as being “forged” in communities of practice, where communities of practice are defined as a group that shares a common interest in a particular subject or who collaborate over an extended period of time with a desire to learn from one another and contribute their own experiences to the community (Lave, 1992; Lave & Wenger, 1991). Communities of practice, such as those created intentionally in informal science experiences, provide opportunities for students to learn the shared language and conventions of the field, to explore and enact science identities, and through their social interactions with other members of the community including their peers, teachers and/or mentors, recognize in themselves and be recognized by others for their competence in science (Aschbacher et al., 2010).

While a growing body of research identifies sense of belonging in science as an important factor in academic achievement, most research has been done at the college level (Hurtoda & Carter, 1997; Korpershoek et al., 2020) or about school or school science (Penuel et al., 2024; Parker et al., 2021; Anderman, 1999; Arslan, 2018; Zhang et al., 2018). Little is known about interventions that successfully promote belonging generally, and even less is known about sense of belonging in science specifically. In addition, according to Allen et al. (2021), there is very little consensus about how belonging should be conceptualized and measured. Indeed, Mark Leary, a pioneering researcher of belonging advised, “The most important thing [for the future belonging researcher] is to be careful and precise in the constructs and terms that you use in your work and to be sure that your measures and manipulations map on to those conceptualizations as tightly as possible” (Allen et al., 2021, p. 1150).

Given a lack of research done on adolescents’ sense of belonging in science (outside of the school context), the need to shepherd more high school students toward science majors and careers, and the state of the field regarding the ambiguous nature of the construct, we conducted a study that situated “belonging” in one domain (science) for one age group (high school students).

### Conceptual Framework for the Sense of Belonging in Science Construct

**Figure 2.**
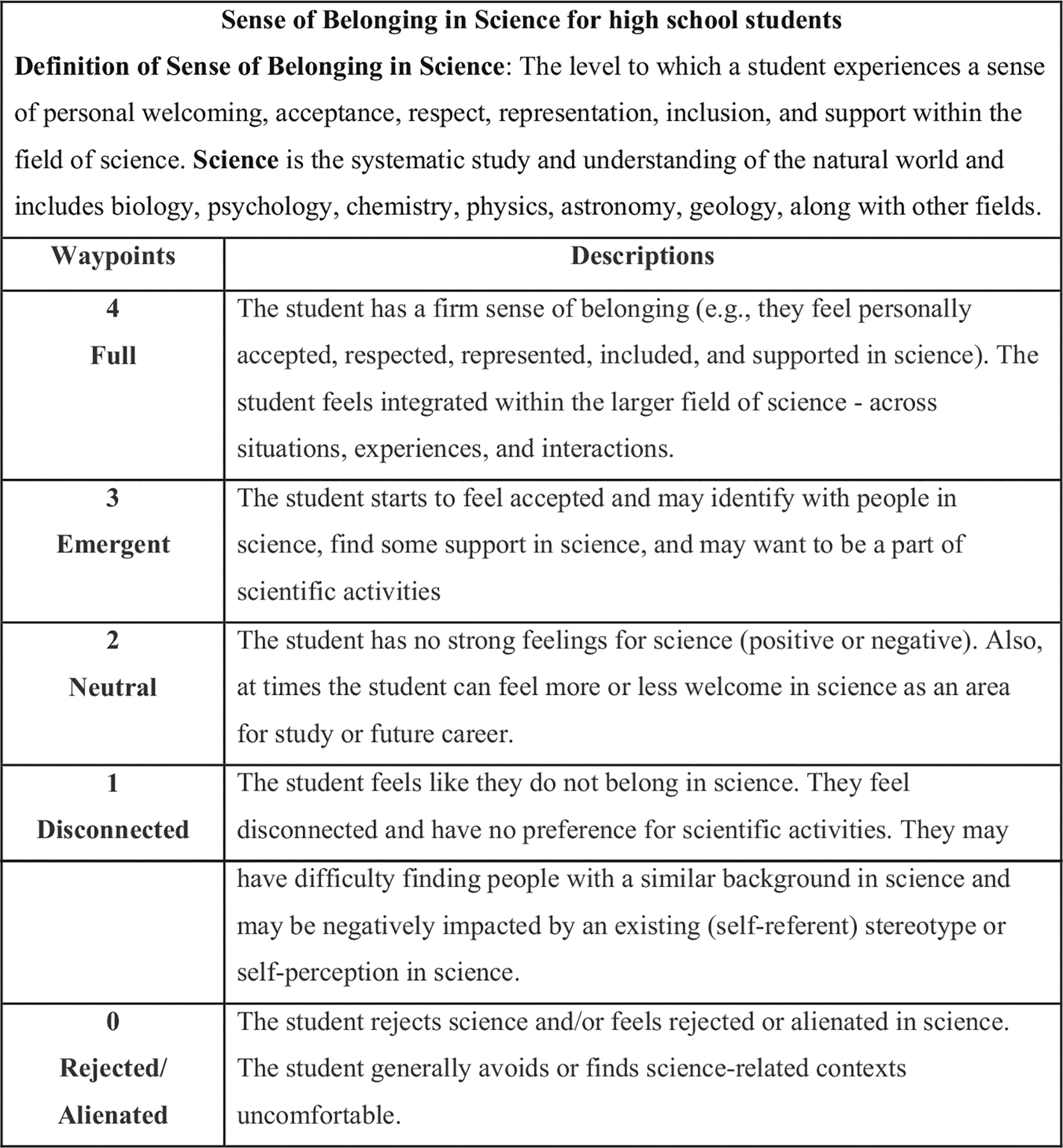
Definition and theoretical continuum of sense of belonging in science.

To begin to understand a high school student’s sense of belonging in science, we propose a progression of stronger and weaker sense of belonging in science based on previous studies about sense of belonging (in general) and sense of belonging in science. This description is critical because it moves the abstract concept of sense of belonging in science toward a clarified concept that can be defined and delineated into a hierarchically based organized structure. Figure 2 provides the theorized structure that contains qualitatively distinct levels or points along the way (e.g., waypoints) that a high school student could move along a continuum. Wilson (2023) suggests that the image in Figure 2 be named a “construct map.” The construct map, the first building block in Wilson’s BAS framework, describes one extreme of an attribute (e.g., knowledge, opinion, belief, etc.) to the other and allows for thinking, reasoning, and attitudes to become visible.

### Context of the Study

The Science and Health Education Partnership (SEP) at the University of California, San Francisco (UCSF) provides a 9-month internship, called the Teen Wellness Connection (TWC) program, where high school students connect with scientists and science educators to develop a day long set of activities, called a “summit,” to engage and benefit their high school peers. Each year, 20 rising high school juniors and five senior leaders (rising seniors who are previous program participants) work with SEP leadership to accomplish several goals. For example, the group designs and hosts a one-day summit yearly for more than 100 high school students. The TWC program is funded by the National Institutes of Health (NIH) and is designed to increase student knowledge of science-related issues, biomedical research, student confidence, relationships with role models in the sciences, and student sense of belonging in science.

Because existing belonging surveys did not target high school students specifically, sense of belonging in science, and/or showed possible technical issues, leaders at SEP collaborated with researchers at the Berkeley Evaluation & Assessment (BEAR) Center at the University of California, Berkeley (UCB) to develop an instrument to measure TWC program participants’ sense of belonging in science. Members at SEP had collaborated with the BEAR Center previously to develop a researcher identity survey (Morell et al., 2024) and were familiar with the instrument development approach, which included working closely with program personnel to iteratively develop the survey over the course of 4–5 years.

## Method

The BEAR Assessment System (BAS), along with the *Standards for Educational and Psychological Testing* (*Standards*, AERA et al., 2014), provides the framework to develop the survey and to inform our validity argument. Using the BAS in conjunction with the *Standards*, we collect reliability evidence and validity evidence based on (test) content, response processes, internal structure, and relation to other variables and evidence of fairness. The BAS was chosen because of the explicit focus on the construct throughout the validation process. By using the BAS as a framework for the study, researchers are able to maintain focus throughout the validation process and streamline the methods to ensure an understanding of the nature and complexity of the attribute of interest.

### Instrument Development

The BAS framework integrates four components into a comprehensive instrument development process. The four components include the construct map, items design, outcome space, and the Wright map (Wilson, 2023). As can be seen in Figure 1, the components are connected, and the process can be integrated through multiple times. See Wilson (2023) for details of the framework.

#### Development of the construct map.

A construct map defines and describes the attribute of interest. It is typically unidimensional and shows the complexity of the attribute as it is expressed by respondents, who have more or less of the attribute. The construct map is the explanation of the theory and forms the foundation of the construct validity endeavor, especially the content and internal structures parts of the validity argument. The construct map is informed empirically by how students express the latent trait and can be mapped to a waypoint using the BAS. The construct map for high school students’ sense of belonging in science is seen in Figure 2.

#### Producing and refining items and their response options.

**Figure 3.**
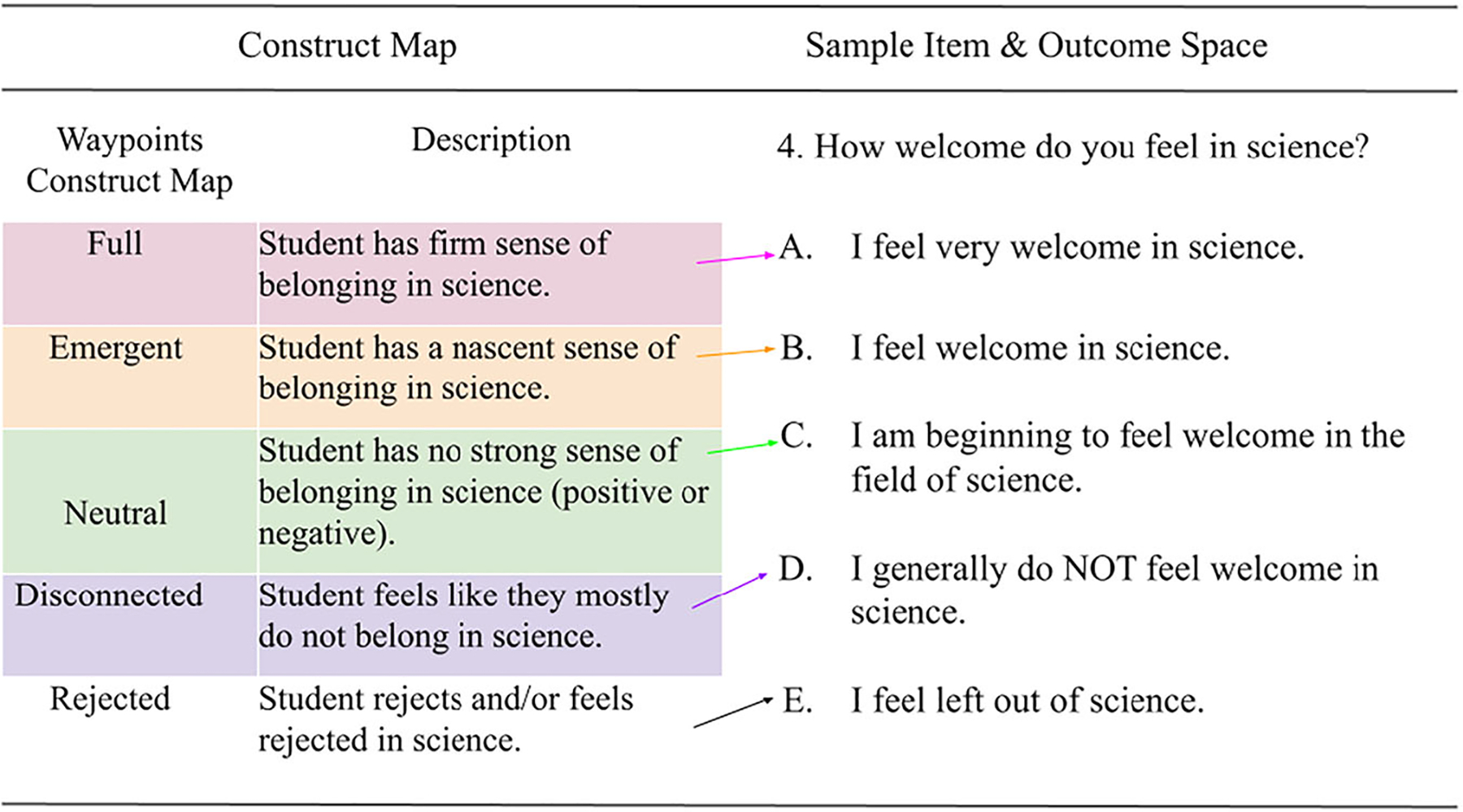
Sense of belonging in science construct map, sample item, and accompanying response options.

After developing a clear understanding of the construct and clarifying it by developing the construct map, the process of item development can begin. Items most commonly take the form of questions on a survey but are not limited to any one format. The response options are designed to accompany the items and provide a way to value or interpret the responses by mapping them back to the construct map. Figure 3 shows an example item with response options for the Sense of Belonging in Science Survey (BiSS) along with their connection to the construct map.

**Figure 4.**
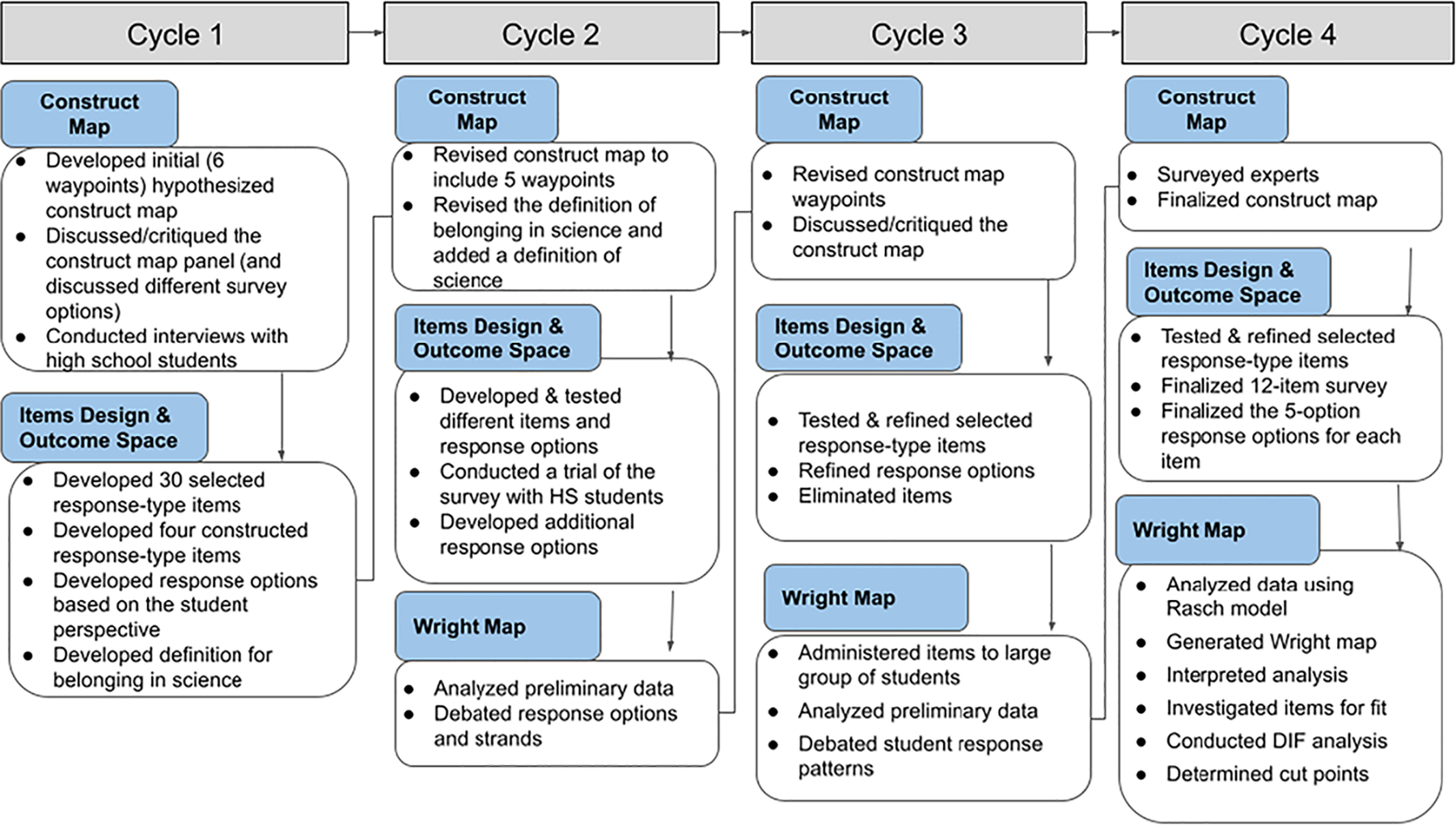
Flow chart of the instrument development process.

Note that each response option maps directly back to the construct map. For example, option A (“I feel very welcome in science.”) maps to the highest waypoint (“Full”) on the construct map (Figure 4). At the highest point on the construct map, the student is said to have a firm sense of belonging, including feeling welcome in science.

#### Generating the wright map.

The construct map, items, response options, and student responses were calibrated using the partial credit model (PCM; 1960/1980), a polytomous version of the Rasch family model.

(1) $$\text{Log}\frac{P(X_{\text{ni}}\text{=}x)}{P(X_{\text{ni}}\text{=}x-1)}=\theta_{n}-(\delta_{i}+\tau_{\text{ix}}),$$

where $X_{\text{ni}}$ represents the observed scores for respondent n on item i; θ, δ, and τ denote the respondent’s proficiency on the construct, item difficulty, and step deviation, respectively. The *TAM* package was employed to run the PCM (Adams et al., 2020; Adams & Wu, 2007). A Wright map was generated based on the PCM results as the final component of the BAS.

We chose to analyze the data using the PCM because it is a member of a family of measurement models that all share the possibility of “sample-free item calibration and test-free person measurement” (Wright & Masters, 1982, p. 38). The psychometric model provides a valuable approach to understanding survey validity by focusing on the fit of the individual item and step to the latent variable model (Wright & Masters, 1982). The model transforms raw information (item scores and person estimates) onto a common scale, which yields item calibrations and person ability estimates on the same interval scale. Moreover, in the Rasch family model, the order of item difficulty remains the same across the span of the construct continuum, which is in line with the logic of a construct map (Wilson, 2023).

We also investigated fairness using a differential item functioning (DIF) analysis (Paek, 2009; Paek & Wilson, 2011) for the self-reported variables of gender and English proficiency. Within the PCM, the test for DIF is specified by allowing the overall item difficulty to differ across demographic categories while controlling for the mean differences in the overall construct (Adams et al., 2020). The equation can be expressed

(2) $$\text{Log}\frac{P(X_{\text{ni}}\text{=}x)}{P(X_{\text{ni}}\text{=}x-1)}=\theta_{n}+\gamma_{g}-(\delta_{i}+\eta_{\text{gi}}+\tau_{\text{ix}}),$$

where g denotes the group membership, γ represents the main effect, and η is the interaction effects that quantify the additional difficulty for different groups. A large $\eta_{\text{gi}}$ indicates stronger DIF.

#### Summary of the development process.

To develop the construct map, items, response options, and the Wright map for high school students’ sense of belonging in science, data were collected from eight high school students through exploratory interviews, 45 students through focus groups, 537 students in the calibration sample, and five experts through surveys. Components of the BAS are identified within each cycle of the instrument’s development as shown in Figure 4.

### Samples

#### Student exploratory interview subjects.

Eight high school students (rising juniors) participated in a loosely structured exploratory interview conducted over Zoom (https://www.zoom.com). Students were recruited from a science internship program. Students attended local schools in a large urban school district. Using ideas generated from the interviews with high school students, we conceptualized a construct of sense of belonging in science for high school students and developed items and response options that were theoretically aligned with the construct.

#### Student focus groups and think-aloud interview subjects.

During Cycle 2 and Cycle 3 of development, different groups of students participated in piloting the BiSS and then participated in focus groups to clarify and refine survey items. Twelve TWC participants were also interviewed using a think-aloud protocol to investigate their response processes. Forty-five students participated in focus groups (21 in Cycle 2 and 24 in Cycle 3). Participants were split into groups of 9–12 students per focus group session. Focus group and interview participants were TWC interns (either rising juniors or seniors in high school).

#### Student survey participants—calibration sample.

A diverse group of 537 high school students in the United States comprised our calibration sample. Twenty-four percent of survey respondents said they were 14 or 15 years old, 27% said they were 16 years old, 33% reported being 17 years old, and 16% reported being 18 years old. Most students reported being in either 11th (33%) or 12th (32%) grade, while others reported being in either 9th (14%) or 10th (21%) grade. Forty-eight percent of the surveyed students identified as female, 46% identified as male, and the remaining 6% identified in queer categories, other, or preferred to not answer. Fifty-five percent of students identified as native English speakers, 30% as advanced, 13% as intermediate, and 2% as beginning English speakers.

Students were recruited through a listserv to high school teachers. Students completed the BiSS, a demographic questionnaire, and a survey measuring general belonging (Malone et al., 2012) during class time.

#### Experts.

Five experts were surveyed regarding the contents of the BiSS. Participants were experts in science education, teaching high school science, belonging, and/or sense of belonging in science. One is a learning and instruction professor with expertise in designing STEM+C learning environments in formal and informal settings. One is an accomplished author with publications about identity and belonging, and professor of learning sciences and human development. One is a PhD-trained chemical engineer and physicist, who identifies as a Blaxican scholar-practitioner whose lived experience informs their commitment to equity and belonging in STEM. As a passionate educator and Director of Learning Experiences at a major West Coast science museum, she designs and leads innovative programs that invite learners from all backgrounds to see themselves in science. One is a lecturer and psychometrician engaged in culturally responsive practices. Finally, one is a former high school science teacher. In addition, four of five of the experts identify as being from at least one minoritized group. Each expert was provided an online review form to record their judgments. There were three forms, with each form containing common items plus a subset of items so as not to overwhelm the experts. Experts were asked to comment on the definition, construct map, items, and response options of the BiSS.

### Analyses

For the qualitative data (from exploratory interviews, focus groups, and think-aloud interviews), we used the grounded theory framework (Glaser & Strauss, 1967) as our analysis guide to ensure that any theories about sense of belonging in science were based on empirical evidence. Transcripts were cleaned and then analyzed using a constant comparison data analysis strategy (Corbin & Strauss, 2008; Charmaz, 2014) in which transcripts are read, preliminary hypotheses are developed by making comparisons among responses while drafting and refining an initial theoretical framework iteratively. Two cycles of analysis were necessary to establish convergence.

Quantitative data collected for the calibration sample were analyzed using the PCM. In reporting quantitative results, we present the reliability coefficients of the BiSS and provide evidence concerning internal structure and relations with an external variable, and fairness examination with DIF analysis. Specifically, for internal structure, item fit statistics are first examined using the information-weighted fit (infit), which quantifies how much the patterns of responses are in line with the Rasch family model, with 1 indicating a perfect fit and an acceptable range from .75 to 1.33 (Wilson, 2023). For the validity of the relationship with the external variable, we administered the Malone et al. (2012) General Belongingness Scale (GBS). The GBS is a 12-item survey using a seven-point Likert scale ranging from “strongly disagree” to “strongly agree,” which was administered online. The GBS showed high reliability and validity based on Exploratory Factor Analysis (EFA) and Confirmatory Factor Analysis (CFA) results. The GBS was validated for use with young adults (mean age of 19.3 years, *SD* = 3.1). The GBS’s sample included males (38%) and females (62%), and the ethnicities of the sample included 41% Hispanic, 35% Caucasian, and 24% Other. It consists of two dimensions: acceptance and rejection. While an equivalent instrument for high school students does not exist, the GBS was chosen to investigate convergent validity evidence because of its closeness of content, online administration, and similarity in age of respondents. In our study, Cronbach’s alpha coefficient of GBS is .88.

## Findings

Evidence of reliability, validity (content, response processes, internal structures, and relation to other variables), and fairness regarding the BiSS was collected using the BAS approach (Wilson, 2023).

### Reliability

Using the calibration sample, which included 537 high school students, the estimated reliability for the 12-item BiSS is calculated to be .91 (Cronbach’s alpha). The expected a posteriori (EAP) reliability is also .91, and the weighted likelihood estimate (WLE) reliability reaches .90. The three high reliability coefficients suggest that the BiSS can assess students’ sense of belonging in science with small standard errors.

**Figure 5.**
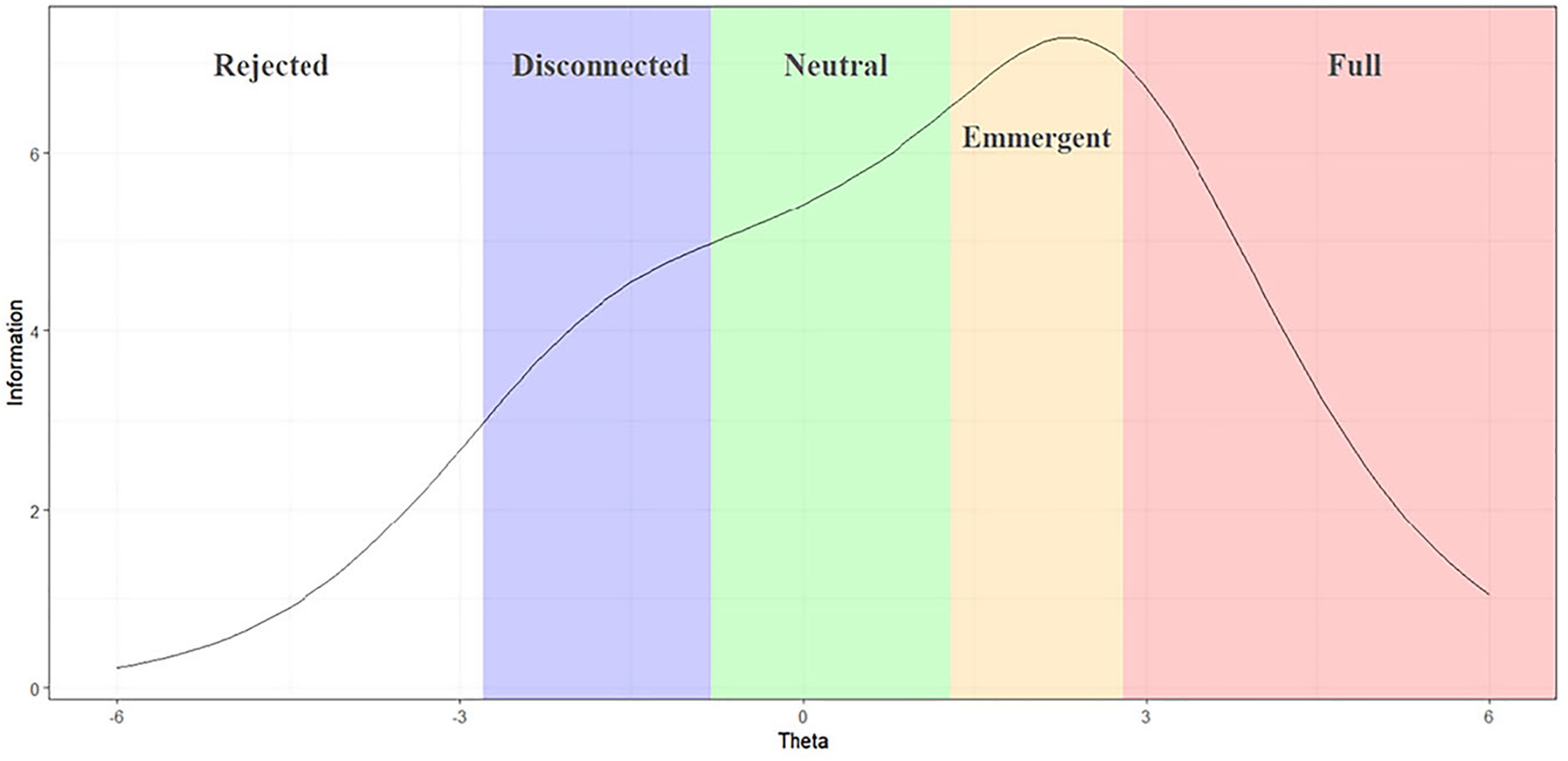
Information curve alongside different waypoints of BiSS.

To further examine the reliability, Figure 5 presents the information curve of BiSS alongside different waypoints. The three waypoints in the middle (i.e., *Disconnected*, *Neutral*, and *Emergent*) obtain higher information than those at the two ends (i.e., *Rejected* and *Full*).

### Validity

#### Validity evidence based on (test) content.

Using exploratory interview data, survey data from the experts, and the PCM analysis of the calibration sample, we collected validity evidence based on test content. Experts provided evidence for the definition and construct map (including waypoints) and the items.

#### Evidence from exploratory student interviews.

Responses from the eight high school students yielded complex descriptions of feelings (such as feeling welcome, included, connected, represented) and structures (such as being a part of a community of individuals thinking like scientists and getting support from knowledgeable adults). Table 1 shows the themes identified through the exploratory interviews along with frequency counts of themes identified during the coding process, and the item stems that were developed to address the themes. The themes were used to develop the item stems as shown on Table 1 and the response options (Figure 3 above shows an example). See the end of the manuscript for the complete survey including all item stems and response options.

One theme mentioned seven times related to doing well academically (e.g., getting good grades or keeping up with the subject matter). While experts indicated that it is important to do well academically, they indicated that the theme stood separate from the sense of belonging construct and recommended it not be included. Therefore, we did not include academic achievement in the table above or in the development of the sense of belonging in science construct. To highlight a few themes, we provide some example quotes from the exploratory interviews here:

#### Belonging.

Belonging is defined by students as feeling included and, in some cases, feeling the absence of a negative emotion. For example, one of the students, Asha,^1^ defined belonging as not being made to feel a certain way, such as “ostracized” or “annoying” and not being in a “negative environment.” Here is an excerpt from her interview^2^: “*I guess not feeling ostracized or annoying. You feel like you have a place in, whatever, since you’re talking about…you just don’t feel like it’s a negative environment for you to be in*.”

Some students defined belonging in terms of feeling comfortable, included, connected, and represented. For representation, students had a variety of ideas. While most indicated that it was important to be seen as an individual, they also identified being represented culturally, ethnically, and/or by gender as helpful to creating a feeling of belonging. For example, Amare said: “*I think that for me personally I feel like I fit in more with people with the same cultural background. Something like that can cause me to feel like if there were people around that did not relate to me culturally like in my background I would not be able to feel like I fit in. It doesn’t have to be a lot, but you know some would help me feel like there’s some representation*…”

Students also said that they felt like they belonged when people were nice, communication was easy, when they felt accepted, and when mentors were helpful and generous with their time. For instance, Malcolm said: “*Everyone was very welcoming [in the informal science program]. They set up meetings with me to get to know me and my mentor even though sometimes the math was hard he spent a lot of time to explain it to me because he knows I’m new to this and he really cares*.”

Students also indicated that having a sense of community was important to feeling a sense of belonging. Here is a quote from Ximena illustrating this point: “*You have a sense of community and you have people around you because I feel like sometimes you can feel like you’re alone, especially if you feel like you don’t really fit in but I think belonging is important because it makes you feel like you’re not alone, and you have people around you that are supportive and help you… the way I describe community is a group of people that kind of come together to help one another*.”

#### Sense of belonging in science.

When prompted to describe what belonging in science means, students often mentioned ideas like the ones that they expressed about belonging in general. However, there were some notable additions. For example, students mentioned feeling accepted, respected, and supported. They also expressed a sense of belonging in science as an internal feeling. For example, Calvin said: “*So probably when somebody/something is belonging in science (putting understanding the basic things aside)…they generally enjoy doing it…any part or specifically a part, but generally it’s just like that feeling you have inside of you—this [science] is what I want to do*.”

Students also indicated that feelings of belonging in science were enhanced when they felt welcomed into a community of like-minded individuals. For example, Akira said: “*Belonging has to do with a feeling of being with your community, like the people that you surround yourself with and I think like that community that I’m part of really helps define my meaning of belonging, because through that program I got to meet a lot of really, really like minded peers, who also wanted to get into college and a lot of really supportive mentors and staff that taught me to live my best version of myself and give my best efforts*.” Finally, students indicated that a sense of belonging in science is related to having interests and feelings (e.g., passion) similar to scientists and being welcomed. For example, Bianca said: “*There’s not really any criteria that you have to meet. It’s just you have to have a passion for it [science] and as long as you have a passion then people are welcoming, and you can learn science and pursue research…*”

#### Evidence from experts.

Five experts were interviewed regarding the contents of the BiSS. Experts were chosen because they had extensive training and experience in science education, teaching high school science, belonging, and/or sense of belonging in science. Experts were asked to comment on the definition, construct map, items, and response options of the BiSS. Regarding the definition four out of five experts indicated that the definition was clear. The expert, a former high school science teacher, who indicated the definition could be improved said: “*Belonging as a construct can be interpreted in many different ways…so…the definition of it [sense of belonging in science] should be broad*.” The other experts and students participating in interviews and focus groups provided contrasting evidence saying that they appreciated the definition and indicated that providing the definition served to ground their thinking. Two of five experts recommended changing the name of a waypoint to better align with the nature of the description. The middle waypoint was originally named “Ambivalent” but was changed to “Emergent” based on the experts’ feedback that the “label ambivalent suggests feelings of both belonging and non-belonging.” Finally, the experts saw the connection between the theoretical waypoints and the response options for each of the items and indicated the items were relevant to the definition (including the construct map). See Figure 3 above for an example of the theoretical relationship between the waypoints (also called levels) and the item and response options.

#### Evidence from participants taking the student survey.

The Rasch model produces a Wright map, which displays a hierarchy of respondents and items along a continuum. By using the PCM, we can investigate the Thurstonian threshold locations of the response categories and student estimates, which provide evidence for the contents of the construct map. This will be discussed in more detail in the next section.

#### Validity evidence based on internal structure.

**Figure 6.**
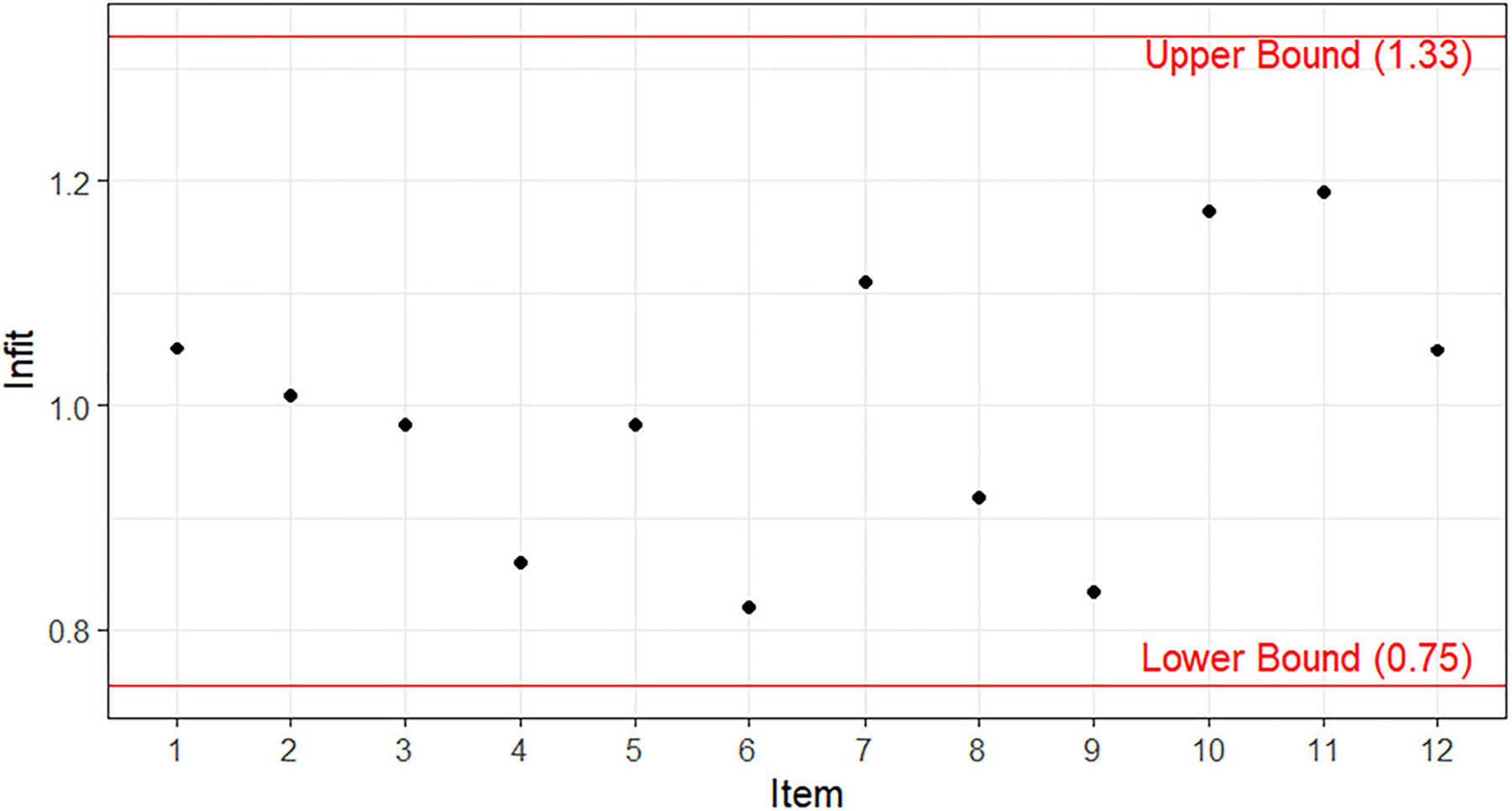
Infit of the 12 BiSS items.

Figure 6 illustrates the infit statistics of the 12 BiSS items. All of them were around 1.00 and fell into the range of .75 and 1.33, indicating that all the items fit well in the PCM at the item level and allowing further examination of the 12 BiSS at the measure level.

**Figure 7.**
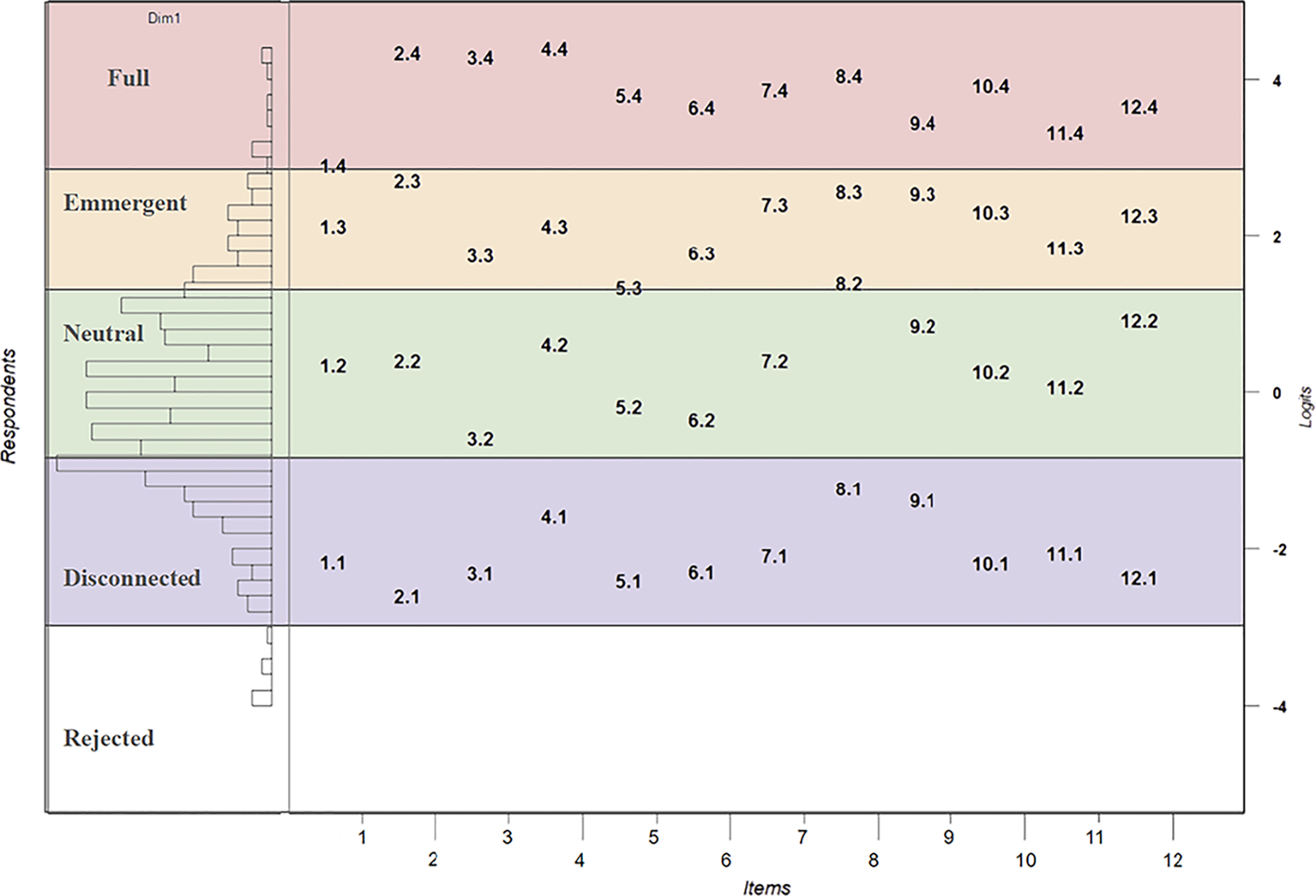
The Wright map of the BiSS.

Figure 7 presents the Wright map. First, the distribution of students’ estimates on the left panel aligns well with the item Thurstonian thresholds on the left panel, indicating that our sample students are suitable for the item calibration. Second, clear cut-points were observed to separate adjacent thresholds across items, which suggests a clear internal structure of the scale and supports the construct map defined at the first step. Third, the span for the *Emergent* waypoint was smaller than the other waypoints, which means less growth is needed for students with an emergent sense of belonging in science to advance to the next waypoint.

#### Validity evidence based on response processes.

From the focus groups and think-aloud interviews, several issues were uncovered and addressed. For example, we found during our first round of focus group sessions that students have varying definitions of “sense of belonging in science” and “science.” This required the development and testing of the definitions. The original definition (e.g., “*Definition: The extent to which students feel personally welcomed, accepted, respected, represented, included, and supported in science*.”) was changed three times before this definition was finalized and adopted:

The early definition was found to be impersonal, so we added “a sense of personal…” to address this critique. We also added a definition of “science” because students had widely varying definitions of science. The interviews and focus groups provided information about how students perceived the survey items and whether those perceptions reflected information on the construct map and aligned with the developers’ intentions.

Based on past research (Carroll et al., 2017; Mishra, 2019; Arslan, 2018), we were curious whether we should include a question about the science teacher perception of the student. Here is the item addressing the teacher’s opinion of the student: *If your science teacher said they think you would do well majoring in science in college, what would you think*? This item ultimately needed to be removed because students who indicated they had a strong sense of belonging in science answered this item in different ways. During focus groups, students said answering this item depends on the teacher, the teacher’s personality, biases the teacher might have, and the student/teacher relationship. Students also said the question did not relate to a sense of belonging in science.

We also intended to include several items about representation. One item asked: *Do you think that scientists in the media represent you?* Focus group participants identified several confusing elements in this question. They had varying ideas about the words “you” and “represent” and almost half of them were not sure the question related to a sense of belonging in science. In addition, one of the experts also mentioned that high school students’ ideas about “media” vary, and the term might be vague. The expert (a former high school teacher) indicated that media could range from a textbook to doctors on TikTok. After two rounds of testing, this question was deleted because student responses varied irrespective of their sense of belonging in science.

Based on data gathered from focus group participants, words on the survey were verified, clarified, and/or changed. For example, focus group participants requested that we clarify by providing examples for two items (Item 1 and Item 5). Item 1 originally asked “How well can you fit into different science groups?” We brainstormed with the focus group participants to develop examples such as “science clubs and classes” to be included in the item’s stem. For Item 5, they also suggested that we be more specific about “qualities of scientists,” so we worked with them and consulted the exploratory interview transcripts to include “curiosity and persistence” as examples in the item’s stem. We also were interested in understanding how students interpreted the term “science community” in Item 3. Students consistently interpreted this as people or groups of people with an interest and/or passion for science. As for changes made based on focus group results, Item 2 originally asked about a student’s “value” to science groups. Focus group participants suggested changing “value” to “contribution,” which students agreed improved the understandability of the item. They indicated they understood contributions to mean ideas about science and science practices.

During think-aloud interviews, students provided rationales for why they chose their answers. For example, one student who described herself as neutral about belonging in science, chose “I am beginning to feel welcome in the field of science” in response to Item 4 [How welcome do you feel in science?] and said: “I don’t think that I would necessarily not be welcome. I just. I think. Okay and in between, like “C” [I am beginning to feel welcome in the field of science],” which indicates the student understands the questions, options, and can choose an option that reflects her thinking. Another student who indicated that he had a strong sense of belonging in science said: “I enjoy science a lot like a subject, so I feel like this one [Choice A: I definitely feel like I belong in science.]” in response to Item 6 [Which statement best describes your sense of belonging in science?].

By the end of the survey development process, the wording for items and response options for the BiSS were deemed understandable, accurate, and clear and the response design in which a question stem is followed by five progressively easier response options was understandable and represented the theoretical waypoints as intended according to the responses during the final focus group session.

#### Validity evidence based on relation to other variables.

Table 2 shows the correlations between the BiSS and the GBS. Note that the second column of Table 2 presents the disattenuated correlation coefficient between the BiSS and the general belonging scale, which consists of two dimensions, that is, acceptance and rejection. We found a positive correlation between the BiSS and the GBS (Acceptance) and a negative correlation between the BiSS and the GBS (Rejection), as anticipated.

## Fairness

We conducted a differential item functioning (DIF) analysis to investigate bias for two variables—gender and language proficiency. All the DIF effects were statistically insignificant, with values ranging from −.05 to .05 logits for genders and −.02 to .03 logits for different language proficiency groups. Therefore, DIF with respect to both variables (gender and language proficiency) was negligible for all items, suggesting the fairness of BiSS.

To summarize, we use the BAS approach to investigate the validity of the Sense of Belonging in Science Survey. Through this approach, we collected the types of validity evidence identified in the Standards (AERA et al., 2014). Specifically, we collected data from experts and students to investigate the test content of the instrument. We collected data from high school students during focus groups and think-aloud interviews for evidence of response processes. We collected survey data to investigate the internal structures of the BiSS; we collected survey data from high school students using the GBS (Malone et al., 2012) to collect evidence based on relations to other variables. We also used the survey data collected to investigate the reliability and fairness of the interpretation and use of the instrument.

## Discussion and Implications

### Overview of the Findings

This research resulted in the first validated survey to measure high school students’ sense of belonging in science. This is significant given that students in high school are already beginning to forge pathways into science and future careers in science and researchers and practitioners have no valid and reliable way to measure the attribute. This study utilizes a construct map approach, provides researchers with a rigorously validated survey that fills an important gap in the literature and enables practitioners to use it to make data-driven decisions to inform the improvement of programs, focus offerings to high school students, and implement interventions to support high school students’ sense of belonging in science.

This study serves as an example of how measurement extends beyond any one psychometric technique or set of techniques in order to ensure a comprehensive approach to instrument design. By centering the investigation on the construct, a variety of data collection methods can be used to discover the complexities of the attribute without veering astray. To demonstrate this, we applied the BAS to investigate and ultimately produce a measure of high school students’ sense of belonging in science. The instrument, a self-report survey containing 12 items with five response options for each item, is based on the construct map, which explicates the theory of how high school students express their sense of belonging in science. Overall, the findings from this research produced strong evidence for the valid, reliable, and fair interpretation and use of the Sense of Belonging in Science Survey as a measurement tool for high school students.

### Reliability

We gathered evidence to ensure the reliability of the survey’s items and person responses. To investigate reliability, we analyzed students’ survey responses from the calibration sample. The estimated reliability of the BiSS items at .91 (coefficient alpha) was high. The person reliabilities (the EAP reliability at .91 and the WLE reliability at .90) were also high. The evidence collected for this study indicates the BiSS produces reliable results.

### Validity

To inform our validity argument, we iterated through four cycles of the BEAR Assessment System to collect qualitative and quantitative data from students through interviews, focus groups, and surveys, and from experts through surveys. This iterative and multi-source approach helps ensure the accuracy of our findings and provides a comprehensive bridging between the theory of sense of belonging in science and the empirical results collected. Multiple sources of empirical evidence were gathered and analyzed to support the quality of the instrument.

From experts, we gathered evidence to ensure the construct map, items, and response options were in alignment and accurately reflected the concept of sense of belonging in science. The Rasch analysis also provided evidence for the content of the material. This process and the resulting PCM results provide strong evidence that the items (and instrument as a whole) can identify students’ locations using the BiSS. For evidence based on response processes, focus groups and think-aloud interviews with students helped clarify wording, definitions, and concepts to ensure high school students understood the definition, items, and response options in the ways intended by developers. Finally, with the positive correlation between the BiSS and the GB Acceptance dimension and the negative correlation between the BiSS and the GB Rejection dimension provides evidence that the BiSS is positively related to similar variables and negatively related to dissimilar variables.

## Summary

This research contributes to the fields of measurement and cognitive science. For the field of measurement, this research serves as an example of how a social cognitive construct can be defined substantively and structured in the same way as an academic construct. This research helps expand the range of applications appropriate for this methodology and also serves as a reminder of the centrality of the construct in the assessment development process. For the field of cognitive science, the construct map extends existing research about sense of belonging beyond school and school science settings (Penuel et al., 2024; Parker, et al., 2021; Anderman, 1999; Arslan, 2018; Zhang et al., 2018). For both cognitive science and measurement, this study serves as an example of how a social cognitive construct can be conceptualized and measured, which in itself is an important contribution.

## Supplementary Material

Appendices

Additional supporting information may be found online in the Supporting Information section at the end of the article.

## Figures and Tables

**Table 1:** Themes from Exploratory Interviews, Frequency of Theme codes, and Item Stems

Themes Indicating a Sense of Belonging in Science*	Frequency of Theme’s Coding	BiSS Item Stems
Belong or fit general, science, or specific area (e.g., biology)	15	1. How well can you fit into different science groups (science clubs, classes, etc.)? 6. Which statement best describes your sense of belonging in science? 7. Which statement describes the sense of belonging you feel every day (e.g., in your community, school, family, friend group, etc.)?
Communication (incl. feeling heard)	23	10. Suppose you want to tell the people in a science-related club about a new scientific finding, what would happen?
Connection	24	3. How connected are you with the science community?
Contribution	15	2. Which statement best describes your contribution to science groups? 12. Suppose you are in a science study group with some classmates, what would happen if you wanted to say something?
Representation	18	11. Which statement best describes how you think about people like you having careers in science?
Similarity to scientist	33	5. To what degree do you have the same qualities (e.g., curiosity, persistence, etc.) that a scientist has?
Welcome (including inclusion and acceptance)	13	4. How welcome do you feel in science? 8. Which of the following best describes you? 9. How included do you feel in science class?

* A total of 8 high school students participated in the exploratory interviews.

**Table 2:** Disattenuated Correlation Coefficients between the BiSS and the GBS

	BiSS	GB	GB Acceptance	GB Rejection
BiSS				
GB	.41**			
GB Acceptance	.46**	.89**		
GB Rejection	−.29**	−.91**	−.63**	

GB = general belongingness = GB Acceptance – GB Rejection; GB Acceptance = the acceptance dimension of GB; GB Rejection = the rejection of GB.

** *p* < .001.

## Footnotes

1 All names have been changed to protect anonymity.

2 Many students used the words “like,” “um,” and “yeah” frequently. These words were omitted without changing the meaning of the quotes.

## Appendix

### Survey of Sense of Belonging in Science

The purpose of this survey is to gain an understanding of your sense of belonging in science. There are no right or wrong answers. Please use the definitions to answer each question. *Belonging in science*: The level to which a student experiences a sense of personal welcoming, acceptance, respect, representation, inclusion, and support within the field of science. *Science* is the systematic study and understanding of the natural world and includes biology, psychology, chemistry, physics, astronomy, geology, along with other fields.

1. How well can you fit into different science groups (science clubs, classes, etc.)?
  - I can fit into almost all science groups easily.
  - I can fit into most science groups if I try.
  - I can fit into some science groups if I try.
  - I can fit into some science groups but feel left out sometimes.
  - I cannot fit into any science groups.

1. Which statement best describes your contribution to science groups?
  - I am an important contributor to science groups.
  - I am a contributor to science groups.
  - I contribute a little to science groups.
  - I contribute nothing to science groups.
  - I contribute negatively to science groups.

1. How connected are you with the science community?
  - I have a strong connection to several people in science.
  - I’m becoming more connected with people in science.
  - I know some people in science but I am not close with them.
  - I do NOT know people in science.
  - I do NOT want to know people interested in science.

1. How welcome do you feel in science?
  - I feel very welcome in science.
  - I feel welcome in science.
  - I am beginning to feel welcome in the field of science.
  - I generally do NOT feel welcome in science.
  - I feel left out of science.

1. To what degree do you have the same qualities (e.g., curiosity, persistence, etc.) that a scientist has?
  - I have many important qualities that scientists have.
  - I probably have some of the same qualities that scientists have.
  - I might or might not have some of the same qualities that scientists have.
  - I probably do not have the qualities that scientists have.
  - I do not have the qualities that scientists have.

1. Which statement best describes your sense of belonging in science?
  - I definitely feel like I belong in science.
  - I sometimes feel like I belong in science.
  - I feel like I could be a part of the science community.
  - I mostly feel like an outsider in science.
  - I feel like I do NOT belong in science.

1. Which statement best describes the sense of belonging you feel every day (e.g., in your community, school, family, friend group, etc.)?
  - I always feel a sense of belonging.
  - I often feel a sense of belonging.
  - I sometimes feel a sense of belonging.
  - I rarely feel a sense of belonging.
  - I always feel left out.

1. Which of the following best describes you?
  - I feel fully accepted as myself when I do science activities.
  - I sometimes feel accepted when I engage in science activities.
  - I am more or less myself when I do science activities.
  - I find it hard to be myself when I do science activities.
  - I cannot be myself while engaging in science activities.

1. How included do you feel in science class?
  - I feel completely included in science class.
  - I mostly feel included in science class.
  - I feel neither included nor excluded in science class.
  - I feel a little left out in science class.
  - I feel left out in science class.

1. Suppose you want to tell the people in a science-related club about a new scientific finding, what would happen?
  - They would encourage me to tell them all I know about the scientific finding.
  - They would welcome the information from me.
  - They might or might not want to hear about the new finding from me.
  - They would probably not be interested in what I had to tell them.
  - They would discourage me from telling them the news.

1. Which statement best describes how you think about people like you having careers in science?
  - There are lots of people like me in science.
  - I see people like me in science.
  - I have seen one or two people like me in science.
  - I do NOT know if people like me have careers in science.
  - I do NOT think I have ever seen someone like me in science.

1. Suppose you are in a science study group with some classmates, what would happen if you wanted to say something?
  - People tend to ask for my ideas.
  - My group members would listen to me.
  - I would be heard if I repeatedly made my point.
  - I do NOT know if I would be heard.
  - No one would hear my ideas.
