# Supplementary material for: Sense of Belonging in Science: A Focus on the Construct
Source: J Educ Meas. Author manuscript; Available in PMC 2026 May 26. (PMC13193266; doi:10.1111/jedm.70038)
Supplement: Appendices [file NIHMS2175888-supplement-Appendices.pdf]

Supplementary Material

Appendix A. Category Characteristic Curve

I1

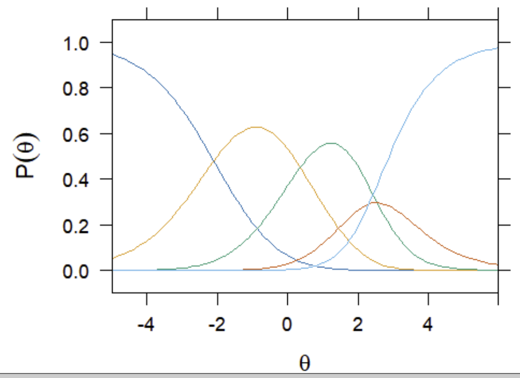

I2

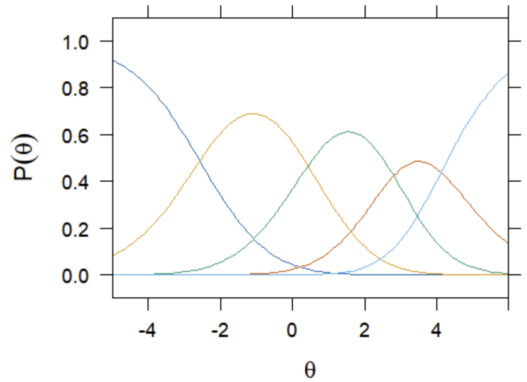

I3

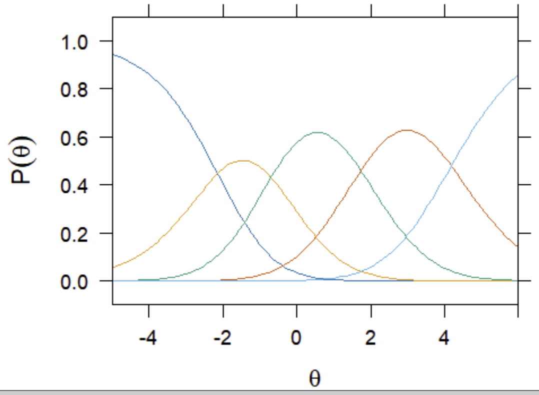

I4

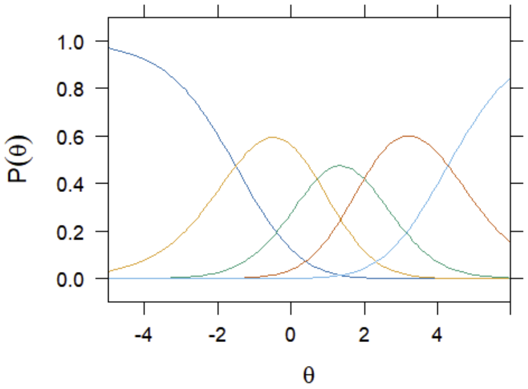

I5

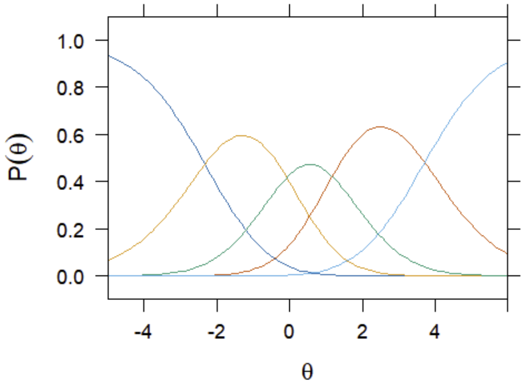

I6

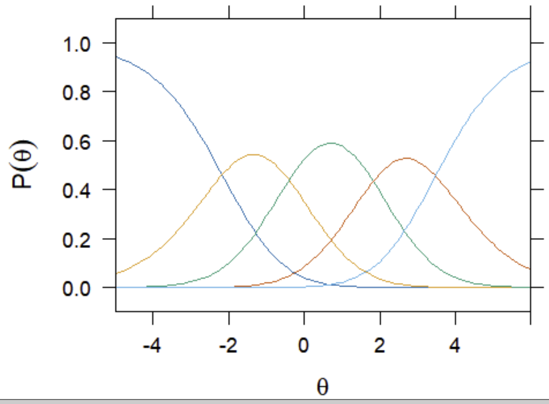

I7

I8

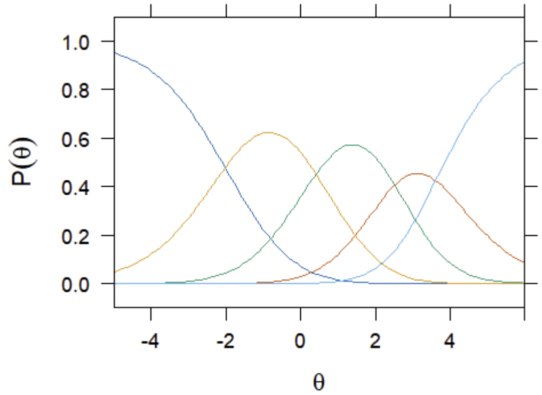

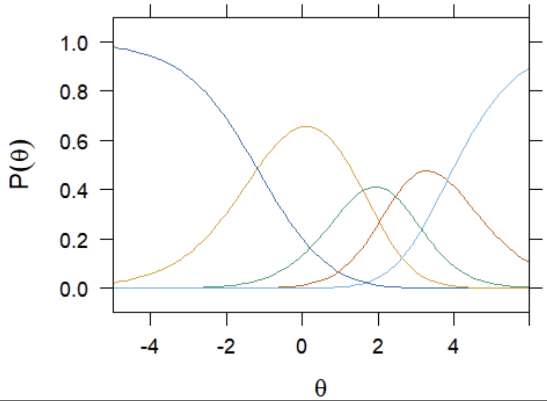

I9

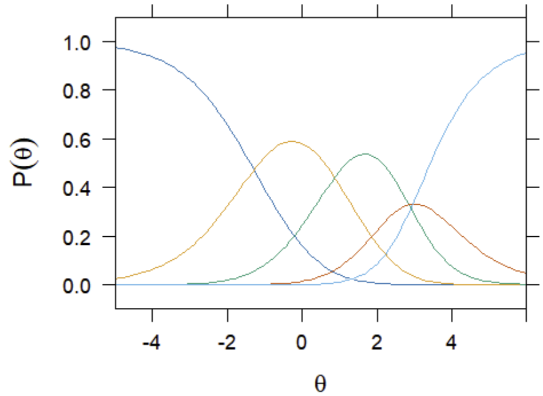

I10

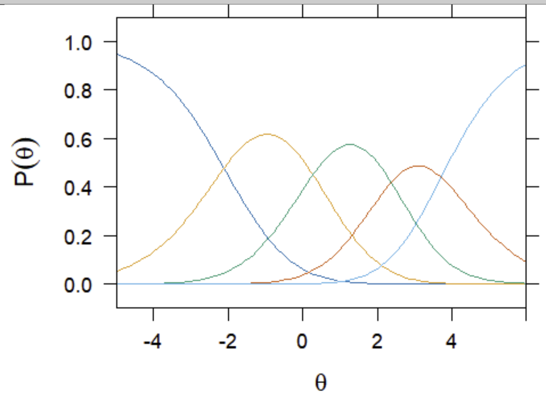

I11

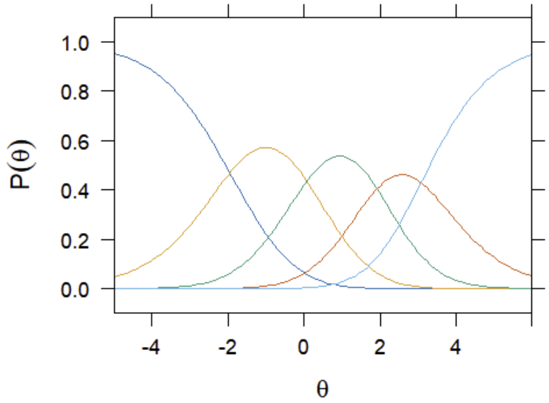

I12

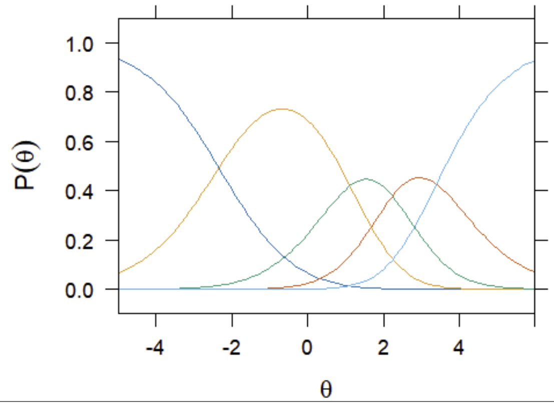

Appendix B. Item Parameters

Table B1.

BiSS Item Thurstone Thresholds

Item	Threshold 1	Threshold 2	Threshold 3	Threshold 4
1	-2.176	0.329	2.114	2.888
2	-2.619	0.393	2.695	4.326
3	-2.326	-0.599	1.738	4.262
4	-1.605	0.601	2.118	4.377
5	-2.422	-0.199	1.313	3.786
6	-2.309	-0.359	1.771	3.630
7	-2.100	0.382	2.393	3.852
8	-1.239	1.369	2.553	4.036
9	-1.391	0.839	2.528	3.431
10	-2.195	0.248	2.287	3.911
11	-2.065	0.051	1.831	3.311
12	-2.374	0.906	2.249	3.635

Table B2.

BiSS Item Parameters

Item	beta	tau.Cat1	tau.Cat2	tau.Cat3	tau.Cat4
1	0.782	-2.881	-0.421	1.767	1.535
2	1.198	-3.771	-0.768	1.613	2.925
3	0.769	-2.913	-1.468	0.966	3.415
4	1.373	-2.881	-0.661	0.629	2.913
5	0.619	-2.948	-0.700	0.554	3.094
6	0.683	-2.852	-1.085	1.146	2.792
7	1.131	-3.152	-0.719	1.391	2.480
8	1.680	-2.861	-0.077	0.786	2.152
9	1.346	-2.637	-0.481	1.506	1.611
10	1.062	-3.175	-0.788	1.313	2.650
11	0.781	-2.732	-0.706	1.139	2.299
12	1.104	-3.447	0.015	1.139	2.293

Appendix C. Residual Analysis:

We ran Principal Component Analysis (PCA) on the residuals from the PCM. Results showed that the eigen value of the first component was 1.72 and it explained only 15.60% of the total variance, which indicates that no major meaningful patterns exist in the residuals.

Table C1 presents the Q3 statistics based on item residuals of BiSS items, The average Q3 is -0.08, and the largest absolute value is 0.23, which suggests no severed local dependence in the residuals.

Table C1.

Q3 Statistics of the BiSS items

item1	item2	Q3	aQ3	p	p.holm
I5	I6	0.153	0.236	0.000	0.000
I9	I12	0.099	0.182	0.000	0.003
I3	I6	0.096	0.179	0.000	0.004
I2	I3	0.084	0.167	0.000	0.011
I8	I9	0.071	0.154	0.001	0.033
I3	I8	-0.233	-0.150	0.001	0.045
I2	I10	-0.227	-0.144	0.001	0.074
I1	I5	-0.223	-0.140	0.002	0.096
I6	I12	-0.222	-0.139	0.002	0.108
I10	I12	0.051	0.134	0.003	0.150
I1	I11	-0.212	-0.129	0.004	0.211
I2	I9	-0.205	-0.122	0.006	0.341
I3	I12	-0.201	-0.119	0.008	0.427
I1	I2	0.036	0.118	0.008	0.427
I4	I11	-0.201	-0.118	0.008	0.427
I3	I9	-0.199	-0.117	0.009	0.459
I1	I4	0.033	0.116	0.009	0.468
I1	I12	-0.195	-0.112	0.012	0.578
I6	I11	-0.194	-0.111	0.013	0.613
I3	I5	0.025	0.108	0.016	0.740
I2	I6	0.022	0.105	0.019	0.853
I4	I6	0.020	0.103	0.021	0.942
I6	I7	-0.186	-0.103	0.021	0.942
I8	I12	0.019	0.102	0.022	0.959
I3	I4	0.013	0.096	0.032	1.000
I1	I8	0.009	0.092	0.040	1.000
I5	I9	-0.174	-0.091	0.041	1.000
I4	I9	0.002	0.085	0.057	1.000
I8	I11	-0.164	-0.082	0.069	1.000

I3	I10	-0.162	-0.079	0.078	1.000
I7	I9	-0.005	0.078	0.083	1.000
I7	I11	-0.008	0.074	0.097	1.000
I6	I9	-0.156	-0.073	0.104	1.000
I3	I7	-0.156	-0.073	0.104	1.000
I6	I8	-0.014	0.069	0.122	1.000
I4	I12	-0.152	-0.069	0.124	1.000
I5	I12	-0.150	-0.067	0.136	1.000
I1	I9	-0.017	0.066	0.140	1.000
I6	I10	-0.145	-0.062	0.164	1.000
I11	I12	-0.022	0.061	0.174	1.000
I1	I7	-0.143	-0.060	0.178	1.000
I2	I12	-0.143	-0.060	0.181	1.000
I5	I8	-0.139	-0.056	0.208	1.000
I2	I5	-0.027	0.056	0.214	1.000
I7	I10	-0.136	-0.053	0.239	1.000
I10	I11	-0.034	0.049	0.274	1.000
I2	I7	-0.130	-0.047	0.296	1.000
I7	I12	-0.036	0.047	0.296	1.000
I4	I5	-0.128	-0.045	0.316	1.000
I2	I11	-0.119	-0.037	0.414	1.000
I5	I10	-0.119	-0.036	0.425	1.000
I3	I11	-0.116	-0.033	0.464	1.000
I9	I10	-0.056	0.027	0.542	1.000
I5	I7	-0.105	-0.023	0.614	1.000
I1	I10	-0.103	-0.020	0.654	1.000
I1	I3	-0.063	0.020	0.658	1.000
I2	I8	-0.065	0.017	0.697	1.000
I5	I11	-0.066	0.017	0.709	1.000
I9	I11	-0.067	0.016	0.720	1.000
I4	I10	-0.098	-0.015	0.730	1.000
I4	I7	-0.068	0.015	0.744	1.000
I2	I4	-0.089	-0.007	0.884	1.000
I4	I8	-0.077	0.006	0.900	1.000
I7	I8	-0.078	0.004	0.921	1.000
I8	I10	-0.087	-0.004	0.927	1.000
I1	I6	-0.086	-0.003	0.940	1.000
